# Evaluation of Platelet-Rich Plasma Therapy for Peripheral Nerve Regeneration: A Critical Review of Literature

**DOI:** 10.3389/fbioe.2022.808248

**Published:** 2022-03-01

**Authors:** Sulong Wang, Xilin Liu, Yueshu Wang

**Affiliations:** Department of Hand Surgery, China-Japan Union Hospital of Jilin University, Changchun, China

**Keywords:** peripheral nerve regeneration/repair, platelet RICH plasma therapy, schwann cell, wallerian degeneration, critical review

## Abstract

Peripheral nerve injury (PNI) is a common disease in clinic, and the regeneration process of peripheral nerve tissue is slow, and patients with PNI often suffer from the loss of nerve function. At present, related research on the mechanism of peripheral nerve regeneration has become a hot spot, and scholars are also seeking a method that can accelerate the regeneration of peripheral nerve. Platelet-rich plasma (PRP) is a platelet concentrate extracted from autologous blood by centrifugation, which is a kind of bioactive substance. High concentration of platelets can release a variety of growth factors after activation, and can promote the proliferation and differentiation of tissue cells, which can accelerate the process of tissue regeneration. The application of PRP comes from the body, there is no immune rejection reaction, it can promote tissue regeneration with less cost, it is,therefore, widely used in various clinical fields. At present, there are relatively few studies on the application of PRP to peripheral nerve regeneration. This article summarizes the literature in recent years to illustrate the effect of PRP on peripheral nerve regeneration from mechanism to clinical application, and prospects for the application of PRP to peripheral nerve.

## Introduction

Peripheral nerve injury is a common nervous system condition associated with a high disability rate. Currently, the best treatment for nerve injury is restoring nerve continuity through microsurgical tension-free anastomosis or autogenous nerve transplantation. This treatment approach does not improve slow nerve regeneration and incomplete postoperative functional recovery. Thus, regeneration and repair of peripheral nerve injury is the focus of intense research.

Although injured nerve can be reconstructed the continuity, this, however, does not create a suitable microenvironment of nerve regeneration (Fowler et al., 2015). Platelet rich plasma (PRP) is a concentration of autologous platelets that releases various growth factors, hence promoting tissue regeneration. PRP has many applications. For example, in stomatology, a randomized controlled clinical trial showed that PRP and its derivatives prevent alveolar bone atrophy and enhance alveolar tissue regeneration (Ucak Turer et al., 2020). A multicenter, double-blind, randomized controlled trial in foot and ankle surgery showed that PRP injection outperforms traditional glucocorticoid injection in plantar aponeurosis treatment (Peerbooms et al., 2019). In sports medicine, a double-blind randomized control study showed that PRP injection can effectively improve healing of old meniscus injury (Kaminski et al., 2019). In chronic sports injury treatment, it is reported that pain relief and functional recovery of lateral humeral epicondylitis (tennis elbow) patients receiving PRP are better relative to controls (Mishra et al., 2014; Merolla et al., 2017). In joint surgery, local PRP injection effectively relieves pain in patients with knee joint osteoarthritis, promoting functional recovery and its effects are superior to traditional hyaluronic acid (Duymus et al., 2017; Lisi et al., 2018). In ophthalmology, PRP can be used to treat secretory dry eye (García-Conca et al., 2019). PRP is also reported to significantly accelerate wound healing (Mohamadi et al., 2019; Zhang et al., 2019). PRP applications are summarized in Table 1.

**TABLE 1:** Summary of clinical application of PRP.

Application	Intervention	Outcome	References
stomatology	Injectable platelet-rich fibrin with connective tissue graft for the treatment of deep gingival recession defects	prevent alveolar bone atrophy and enhance alveolar tissue regeneration	Ucak Turer et al. (2020)
Foot and ankle	injection	PRP injection outperforms traditional glucocorticoid injection in plantar aponeurosis treatment	Peerbooms et al. (2019)
sports medicine	injection	improve healing of old meniscus injury	Kaminski et al. (2019)
chronic sports injury	injection	pain relief and functional recovery of lateral humeral epicondylitis (tennis elbow) patients receiving PRP are better	Mishra et al. (2014), Merolla et al. (2017)
joint surgery	injection	local PRP injection effectively relieves pain in patients with knee joint osteoarthritis, promoting functional recovery	Duymus et al. (2017), Lisi et al. (2018)
ophthalmology	external application	PRP can relief the symptoms of secretory dry eye	García-Conca et al. (2019)
wound healing	Injection and external application	It can significantly accelerate wound healing, promote vascularization and granulation tissue regeneration	Mohamadi et al. (2019), Zhang et al. (2019)

In conclusion, decades of clinical practice show that PRP promotes tissue repair and regeneration. Moreover, this approach does not need special equipment and training, and is cost effective, making it of great value in regenerative medicine (Etulain, 2018). Here, we comprehensively review the effects of PRP on peripheral nerve regeneration, the mechanisms underlying PRP promotion of peripheral nerve regeneration, and PRP clinical applications for peripheral nerve regeneration.

## Peripheral Nerve Regeneration

Peripheral nerve injuries are clinically prevalent and injury types can be classified as open nerve injuries, such as glass injury, machine crush injury, sharp instrument injury, and crush injury, or and closed injuries like nerve compression disease (such as carpal tunnel syndrome and cubital tunnel syndrome). Each injury mechanism causes varying damage to the nerve tissue, surrounding soft tissue, and its blood supply, hence recovery also differs. Thus, injury type significantly affects peripheral nerve recovery. Age, gender, patient’s health, and underlying diseases are also important factors in peripheral nerve regeneration (Kuffler and FOY, 2020). Nerve injury triggers Wallerian degeneration in the distal nerve. The distal myelin sheath, Schwann cells, and axons disintegrate and cell debris is phagocytized by macrophages. Axon and myelin phospholipids degeneration leaves Schwann cells in the basal lamina, which encapsulate nerve fibers. The Schwann cell linings in the basal layer are called endoneurial tubes or Bungner bands. Axon regeneration occurs along the Bungner zone. Schwann cells are critical in the process of nerve regeneration, from Wallerian degeneration to axonal myelination. Schwann cells regulate nerve regeneration by interacting with various cells in the regeneration microenvironment. This section discusses peripheral nerve regeneration through Wallerian degeneration, axon regeneration, and Schwann cells (Fawcett and KEYNES, 1990).

### Wallerian Degeneration

Nerve damage close to the cell body causes neuron death. When damage is far from the cell body, nerve fibers regenerate. The axon disintegration and nerve demyelination at the distal end are called Wallerian degeneration. Wallerian degeneration is a unique form of axonal degeneration and a necessary process after peripheral nerve injury. Upon nerve damage, Schwann cells and neuroendymal fibroblasts undergo gradual apoptosis and 2 days later, axons degenerate due to Ca^2+^ and Na^+^ release. Subsequently, injured axons release neuropeptides like substance P and calcitonin gene-related peptide (CGRP) to cause neurovascular dilatation. Neural vessel expansion and chemotaxis due to the release of monocyte chemoattractant protein-1 by Schwann cells promotes migration and recruitment of macrophages (Toews et al., 1998). Macrophages may then phagocytize the axon and myelin sheath debris, creating a good environment for nerve regeneration. Macrophages may also secrete vascular endothelial growth factor (VEGF-A), promoting new blood vessel formation in the nerve. At this stage, intact Schwann cells release nerve growth factor (NGF), ciliary neurotrophic factor (CNTF), brain-derived neurotrophic factor (BDNF), and glial derived nerve growth factor (GDNF), thus stimulating formation of new Schwann cells. Schwann cells, in an interlaced and linear arrangement, form a Bungner band, which directs subsequent axon regeneration.

### Peripheral Nerve Regeneration

Upon peripheral nerve injury, damaged axons, non-neuronal cells, Schwann cells, neuroendymal fibroblasts, and macrophages constitute the nerve regeneration microenvironment. For instance, during vein bridging after sciatic nerve defect, fibrin deposition occurs first in the nerve space after injury. Next, red blood cells, granulocytes, platelets, macrophages, and endothelial cells infiltrate the nerve root space (Min et al., 2021). Macrophages form the largest proportion of this cell population, while vascular endothelial cells have the smallest. Peripheral nerve cells also react rapidly after injury, migrating from the nerve stump to the nerve space. Peripheral nerve cells migration follows the fiber deposition network, forming circular channels to control migration of nerve fibroblasts and endothelial cells. Inflammatory cell numbers decrease gradually with time. VEGF-A release by macrophages promotes formation of new blood vessels at the two nerve ends. Newly generated blood vessels are consistent with the long axis of the nerve. Schwann cells migrate on the basis of neovascularization and form schwann cell cords (Cattin et al., 2015). The regeneration of Schwann cells was guided by SCs and is divided into two slow stages (86 μm/day). In the initial stage, axon regeneration does not occur simultaneously with SC regeneration. In the second stage, the speed was 433 μm/day, and the migration and axon took place simultaneously (Torigoe et al., 1996). Netrin1/DCC between Schwann cells and axons is a key signal for axon regeneration. Netrin, a family of extracellular adhesin-related proteins has a key role in nervous system axonal guidance. Schwann cells guide and promote nerve regeneration *via* Netrin1 binding to DCC receptor on axons (Dun and PARKINSON, 2017). Nerve regeneration of the defect model requires deposition of fibrin and the formation of 3D scaffolds for cell crawling. Over time, fibrin scaffolds are gradually replaced by regenerated axons and SC cords.

### Role of Schwann Cells in Axonal Regeneration After Peripheral Nerve Injury

SCs and non-neuronal cells like macrophages, fibroblasts, and lymphocytes cooperate in nerve repair. SCs interact with various cells to regulate nerve regeneration. Schwann cells may be myelinated or unmyelinated (Remak cells), with myelinated Schwann cells having a larger diameter than Remak cells, which are mainly distributed around small sensory and autonomic axons (Stratton and SHAH, 2016). Upon peripheral nerve injury (such as transection injury), macrophages are the main non-nerve cell type present. Additionally, Schwann cells remaining in the nerve stump also promote macrophages recruitment by releasing cytokine monocyte chemoattractant protein-1 and leukemia inhibitory factor (Kroner et al., 2014). Macrophages have two phenotypes; M1 macrophages that are proinflammatory, and the anti-inflammatory M2 macrophages that promote tissue regeneration. Monocyte chemotactic factor-1 release by SCs promotes macrophage polarization towards the M2 phenotype. However, both macrophage types are essential for peripheral nerve regeneration. VEGF is the main factor released by macrophages. In hypoxic condition, macrophages adapt to the microenvironment by secreting VEGF-A, which stimulates blood vessel formation in the direction of nerve regeneration. These newly polarized vessels promote and guide Schwann cell proliferation and cross the nerve stump. The growth arrest-specific gene 6 (Gas6) expressed by macrophage is reported to bind to TYRO3, a Tam tyrosine kinase receptor expressed in SCs, to improve and accelerate human axon myelin formation (Stratton et al., 2018). Macrophage-expressed Slit-3 binds to Robo1 on the surface of migrating Schwann cells to keep SCs in the neural space. Migration of neurofibroblasts and SCs promotes SC arrangement and formation of strand through ephrinb2-ephb signal interaction (Parrinello et al., 2010). Upon nerve injury, damaged SCs and axons downregulate myelin gene expression in Schwann cells and activate a series of repair support functions. Increased c-jun transcription factor levels in SCs leads to the recoding, dedifferentiation, and transformation of SCs into repair type SCs (Jessen and MIRSKY, 2016). Repair type SCs enhance recruitment of inflammatory cells (macrophages, neutrophils) in nerve injury sites by releasing a series of pro-inflammatory cytokines. These cell types also release a variety of neurotrophic factors like NGF, BDNF, and fibroblast growth factor (FGF). Repair SCs bridge the nerve stump by forming a bungner band. Moreover, fibroblasts can interact with repair type SCs and secrete extracellular matrix molecules like tendon protein and tenascin-C (an ECM glycoprotein). Some ECM molecules can enhance SC migration and axonal proliferation by binding to integrin on the surface of SCs (Zhang et al., 2016; Qu et al., 2021).

## Platelet Rich Plasma Promotes Peripheral Nerve Regeneration

As an autogenous active substance, PRP has high levels of platelet, growth factors, leukocytes, fibrin, and various bioactive factors, including fibronectin, osteonectin, and vitronectin). These components are critical for tissue repair. First, platelet activation can stop bleeding and release various growth factors. Different growth factors influence all aspects of tissue repair. White blood cells help clear local pathogens and necrotic tissues. Fibrin can form a three-dimensional network structure in injured tissue and provide a scaffold for tissue regeneration.

There are six evidences of PRP’s potential to promoting nerve regeneration: 1) neuroprotection and prevention of neuronal apoptosis, 2) stimulation of vascular regeneration, 3) promotion of axonal regeneration, 4) regulation of inflammatory response in the microenvironment, 5) alleviation of nerve collateral muscle atrophy, and 6) improvement of human nervous system parameters (Sánchez et al., 2017a). Here, PRP effects on SCs, regulation of inflammatory environment, and axon regeneration were extensively introduced.

### Platelet Rich Plasma

PRP refers to the platelet concentrate extracted from autologous blood by centrifugation. PRP also contains various growth factors, including PDGF, TGF-β, VEGF, EGF, and IGF-1, which are secreted *via* platelet degranulation. These growth factors mainly exist in platelet alpha granules. The basic components of PRP was list in Table 2.

**TABLE 2:** Summary of the components of PRP.

Contents	Functions	Stages
Platelet	Hemostasis	Debridement stage
White cell	Clear local pathogens and necrotic tissues	
Fibrin	Form a 3D network structure in injured tissue and provide a scaffold for tissue regeneration	
Leukocyte	Local anti-infection and inflammatory regulation	Inflammatory stage
TGF-β	Regulation of inflammatory	
PDGF	Angiogenesis stimulation; mitogenesis; macrophage activation	
VEGF	Vasculogenesis; increase perifollicular vessel size during the anagen growth phase	Reconstruction stage
Cytokines	Promote regeneration process	
EGF	Angiogenesis stimulation; cell growth, proliferation	
HGF	Angiogenesis stimulation	
FGF	Angiogenesis stimulation	
IGF-1	Angiogenesis stimulation	

Upon platelet activation, alpha granules fuse with the cell membrane, triggering degranulation and growth factor release. PRP also contain high levels of white blood cells (neutrophils, monocytes, lymphocytes), which phagocytize invading pathogens and foreign bodies. The fibrin in PRP forms a three-dimensional network structure after platelet activation and aggregation, providing a scaffold for repairing the tissue. According to this principle, Dohan and Choukroun proposed the 2nd generation of PRP, namely Plantelet Rich Fibrin (Dohan et al., 2006). Dohan believes that blood can coagulate naturally during centrifugation without anticoagulant, and fibrin can produce a more suitable structure in such a slow polymerization process. In 2014, Choukroun developed advanced PRF (A-PRF) *via* centrifugation at 1,500 rpm for 14 min (Ghanaati et al., 2014). Compared with previous PRF, this PRF is looser and has greater porosity, which is conducive to the diffusion of oxygen and nutrients, and cell proliferation. Cells and growth factors can chemically bond with fibrin, thus minimizing cells and growth factor loss during production. In 2017, Choukroun et al. introduced injectable-PRF (I-PRF) obtained by centrifugation at 700 rpm/60G, for 3 min. This PRF has great plasticity relative to traditional PRF, surmounting limitations of gel-like PRF application (Miron et al., 2017). Third stage PRP is concentrated growth factor (CGF), which was proposed by Sacco (unpublished data). CGF is obtained *via* differential centrifugation from anticoagulant-free venous blood. This centrifugation method fully activates platelets, releasing more growth factors from alpha granules and producing growth factor-rich, CD34^+^ cells. CGF can be prepared in liquid, loose gel, and gel states according to different blood vessels. The classifications of PRP is shown in Table 3.

**TABLE 3:** Summary of classifications of PRP (Huang et al., 2017).

Classification	Content	Characteristics	Biomechanical properties
P-PRP	Platelets with low-density fibrin network after activation, without leukocytes	Liquid solution or gel	Dissolves quickly
L-PRP	Platelets with low-density fibrin network and leukocytes	Liquid solution or gel	Dissolves quickly
P-PRF	Platelets with high-density fibrin network and without leukocytes	Gel	Solid gel, cannot be injected
L-PRF	Platelets and half of the leukocytes, with a high-density fibrin network	Gel or blood clot	Solid gel or blood clot, cannot be injected
A-PRF	Platelets with high-density fibrin network and high concentration growth factor	Gel	Solid gel, cannot be injected
CGF	High concentration of growth factor	Liquid solution	Dissolves quickly

### Regulation of Platelet Rich Plasma on Schwann Cells

SCs have important roles in peripheral nerve injury repair and regeneration. SC proliferation and migration bridges the nerve stump to form the Bungner band, promotes axon regeneration by secreting various active substances, including neurotrophic factors, and can differentiate into myelin sheath at the mature stage of nerve regeneration. PRP contains a high concentration of growth factors, which promote SC proliferation and migration. A recent *in vitro* study of the effects of P-PRP on SCs suggests that platelet-derived growth factor (PDGF-BB) and insulin-like growth factor-1 (IGF-1) may be the main cytokines affecting SC proliferation and migration. In that study, PDGF-BB and IGF-1 antibodies counteracted the proliferation and migration of human SCs cultured in the presence of P-PRP (Pereira et al., 2020). This study also confirmed the positive effect of P-PRP on nerve growth factor secretion by SCs and indicated that low P-PRP concentration (5%) was better. The effect of P-PRP on the biological behavior of SCs was also evaluated *in vitro*. Primary SCs were cultured in different P-PRP concentrations. Cell growth assays and flow cytometry were used to assess their proliferation. RT-qPCR and ELISA were used to evaluate P-PRP effects on NGF and glial cell line derived neurotrophic factor (GDNF) production by SCs. Cell migration was evaluated by micrometastasis assay. These assays found that P-PRP significantly affects Schwann cells behavior, with proliferation and migration highest at 2.5–20% P-PRP (Zheng et al., 2016). An analysis of the effects of P-PRP and PPP (platelet poor plasma) on spiral ganglion neurons found that P-PRP-treated spiral ganglion cells had a good survival rate and that their proliferation rate was greater than that of the blank control group (Stolle et al., 2018). Wang et al. (2020) reported a L-PRF autologous scaffold for peripheral nerve injury. the SCs proliferation and secretion of neurotrophic factors and its anti-inflammatory effect *in vitro* were evaluated. The results indicated that L-PRF can increase SC proliferation, neurotrophic factors secretion, and suppress SC PG-LPS-induced inflammatory responses. Qin et al. (2016a) examine the effects of CGF on the migration, proliferation and neurotrophic factor secretion of SCs. These researches proof that the CGF could significantly promote the proliferation and neurotrophic factor (NGF & GDNF) secretion of SCs. And it could also promote SCs migration partly through the integrin beta 1-mediated activation of the focal adhesion kinase pathway (Qin et al., 2016b).

### Regulation of Platelet Rich Plasma on Inflammatory Cells

Peripheral nerve injury repair involves various inflammatory cells, including macrophages. Macrophages fall into two phenotypes-the classical activated macrophage M1 and selectively activated macrophage M2 (Delavary et al., 2011). The proinflammatory M1 macrophages mainly express nitric oxide synthase (NOS) and are involved in antigen presentation. Normal tissues get damaged by inflammatory reactions. M2 macrophages mainly express arginase type 1 (Arg-1), secrete consistent cytokines, and suppress immune response, playing critical roles in tissue repair. Early stage nerve injuries are mainly associated with M1 macrophages, which phagocytize the myelin sheath and other cell debris upon distal nerve disintegration. Thus clearing substances affecting nerve regeneration and creating a good environment for nerve regeneration. In the late stage of inflammatory reaction, M2 macrophages are the main phenotype and they promote fibrosis and tissue regeneration.

Schwann cells is known to express particular growth factors at extremely high levels post-injury. When assessing peripheral nerve injury, the expression of growth factors such as GDNF, FGF5 and Shh, but not classic M2-inducing cytokines (IL4, IL10, IL13), seem to dominate the nerve during peak stages of debris clearance (Painter et al., 2014). Schwann cells do not secrete high levels of classic M2-associated cytokines, but are potent inducers of M2-phenotypes in macrophages and that these macrophages promote axonal outgrowth. It has also been shown, recently, using Schwann cell transplant in PNI, that macrophage modulation may be one of the contributing mechanisms leading to enhanced regeneration following Schwann cell transplant therapy (Stratton et al., 2016).

As an autologous platelet concentrate, PRP growth factors regulate inflammatory response after injury, which can promote macrophage aggregation, enhancing phagocytosis and antigen presentation. PRP also promotes transformation into M2 macrophages, hence promoting tissue repair and regeneration. PRP can also promote transformation into M2 macrophages by activating JK1/3-STAT6 pathway, hence promoting tissue healing *via* macrophage activation (Lana et al., 2019). *In vivo* studies (Nishio et al., 2020)found that PRP promotes macrophage aggregation during tissue healing, and the effect of PRP on inflammatory cells depends on the composition and concentration of white blood cells, platelets, and red blood cells in PRP. LR-PRP (Leukocyte Rich PRP) can lead to a more obvious inflammatory response, and the aggregation and infiltration of inflammatory cells stimulate tissue repair faster. On the other hand, because LR-PRP more strongly induces metabolism, it can stimulate the proliferation and regeneration of tissue cells faster. A recent *in vitro* study (Wang et al., 2020) using L-PRF as an inducible scaffold for SCs to examine the effects of PRF on SC proliferation, neurotrophic substance secretion, and the anti-inflammatory effect of L-PRF on PG-LPS induced inflammation found that L-PRF influences nerve regeneration and neuroinflammation. Highlighting its potential as an autogenous biological additive for peripheral nerve regeneration.

### Promoting Axonal Regeneration

The vascular system of the peripheral nerve consists of two parts: the peripheral vascular system (the peripheral blood vessels around the perineurium connected to the external vascular network through perforating vessels) and the internal vascular network (the vascular network around the neurointima composed of arterioles and venules capillaries involved in substance exchange in neurons). Nerve tissue are separated from vascular tissue by the vascular nerve barrier, which is comprised of the neuro fascicle and neuro intima. The fascicle mainly offers mechanical connection and due to numerous fibroblasts and myofibroblasts on its membrane, it has a strong anti-tension effect. The main function of nerve intima wrapping axoplasm is to maintain the stability of axonal internal environment and substance exchange. In the study of peripheral nerve model (Caillaud et al., 2019), vascular response is divided into two stages. In the early stage (1st week), the dilation and release of various blood vessels results in vasodilation while the number of blood vessels is unchanged, promoting macrophage recruitment and cell debris debridement. In the 2nd stage, the blood vessel number increases, promoting axonal elongation, cell proliferation, and myelination.

As a platelet concentrate, PRP contains various cytokines that promote regeneration, including VEGF, a powerful angiogenic factor. VEGF is specific to the vascular endothelium, has chemotaxis effects, and promotes the proliferation and migration of the vascular endothelium. VEGF is a crucial modulator of angiogenesis during embryonic development and regeneration of peripheral nerves. Additionally, VEGF promotes neuronal survival and promotes axonal growth. An *in vitro* study of VEGF gene therapy in the treatment of postpartum brachial plexus palsy showed that brachial plexus injury after birth leads to loss of motor neurons. If motor neurons are permanently lost, organ function will also be lost. VEGF is also neuroprotective and can improve motor neuron survival and reduce their susceptibility to ischemic environments (Pereira Lopes et al., 2011; Hillenbrand et al., 2015). An *in vitro* study of the application of VEGF gene therapy to nerve regeneration found a positive correlation between increased vascularization and enhanced nerve regeneration, suggesting that VEGF application may support and promote the growth of regenerated nerve fibers through the combined effects of angiogenesis, neurotrophy, and neuroprotection (Pelletier et al., 2015). Takeuchi et al. Evaluated the promotion of PRP on axon growth, and added neutralizing antibodies against VEGF to the cocultures with PRP. The results indicated that addition of PRP to the cocultures promoted axon growth, and the axon growth was significantly suppressed by the addition of neutralizing antibodies against VEGF (Takeuchi et al., 2012).

**FIGURE 1:**
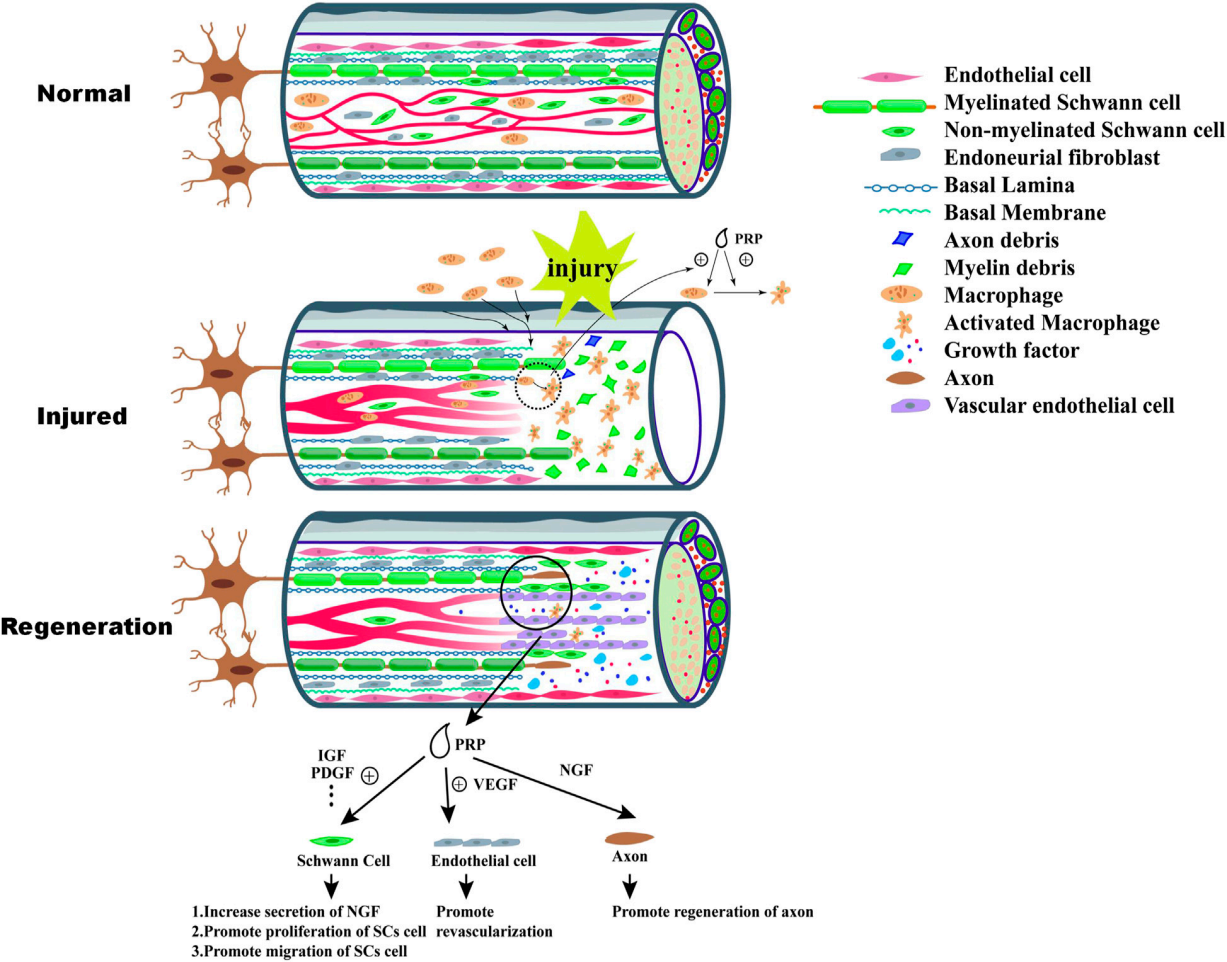
Wallerian degeneration happens after injury of peripheral nerve, which include disintegration of axon and nerve demyelination at the distal end. The debris of nerve tissue are engulfed by macrophages recruited from peripheral blood or nerve tissue, which can provide a suitable environment for nerve regeneration. Monocyte chemoattractant protein-1 (MCP-1) secreted by Schwann cells promote recruitment of macrophages. PRP exert positive effect on recruitment and polarization of macrophage. After Wallerian degeneration, the process of nerve tissue regeneration begin. VEGF-A released by Schwann cell and activated macrophage play a significant role in the process of the formation of neovessels which supply oxygen and nutrients to the formation of “bands of Bünger”. At the same time, intact Schwann cells secrete nerve growth factor (NGF), brain-derived neurotrophic factor and glia-derived neurotrophic factor (GDNF) which stimulate the proliferation of Schwann cell and formation of “bands of Bunger”. As the formation of neovessels, Schwann cells migrate along the vascular which also guide regeneration of axon. PRP contain high concentration of various growth factor which can promote effectively biological activity of Schwann cells, such as migration, secretion of NGF and proliferation. Additionally, high concentration of VEGF in PRP can improve revascularization.

Besides VEGF, brain-derived neurotrophic factor (BDNF) in PRP also promotes nerve regeneration. There is few report to directly demonstrate the positive effect of PRP-derived BDNF on neuron or axon elongation. Because the concentration of BDNF in PRP was low. However, Recent *in vivo* studies show that BDNF promotes the survival and differentiation of neurons as well as endothelial cell survival to maintain blood vessel stability (Lu et al., 2019). Castro reported that the PRP could increased the expression of BDNF in nerve trauma site (Castro et al., 2019). And Zhao’s study further indicated the positive combined effect of PRP and BDNF on axonal remyelination (Zhao et al., 2013). The neural regeneration process and the effect of PRP was summarized in Figure 1.

### Other Relevant Experimental Evidence

For experimental rats, the most widely used model is the nerve defect model because it allows dynamically observation of the process of nerve regeneration. A rat peripheral nerve defect models was used to assess the effect of PRP and hyaluronic acid on peripheral nerve regeneration. The defect was bridged by allogeneic aortic graft, and PRP and hyaluronic acid were added into the aorta. The control group received nerve transplantation. At 12 weeks after operation, morphometric, histological, electromyography and gait analyses showed that axonal and nerve regeneration and the recovery of electromyography and gait in PRP group were better than those in control group (Firat et al., 2016). PRF was transformed into a nerve conduit (PRF-NGC), and a 5 mm-long nerve defect area was established and bridged with a prf-ngc, autologous nerve graft (ANG) and polyurethane nerve conduit. Histological evaluation conducted after 12 weeks showed that the experimental group treated with PRF as the nerve conduit bridging defect was significantly better than the other two experimental groups in terms of myelin sheath thickness and number of regenerated axons. The authors postulated that the nerve conduit made of PRF has great clinical application potential (Huang et al., 2020). Roth et al. (2017) reported that PRF nerve conduit and nerve transplantation have similar therapeutic effects on sciatic nerve defect in rats. Therefore, PRF can be used as a substitute for nerve graft in the treatment of nerve defects (Roth et al., 2017). In addition, PRP combined with acellular nerve allografts (ANAs) has been found with good therapeutic effects in the repair of long nerve defects, and can replace autologous nerve transplantation. Zheng et al. (2014) used the acellular nerve graft to bridge the nerve stump in a rat model of nerve defect, and compared the histological, electrophysiological and neurotrophin expression of PRP, PPP, and autologous nerve across the transplantation groups. Compared with the other two experimental groups, ANA + PRP group had the largest number of axonal regeneration and the highest expression of neurotrophins. In addition, they found that ANA + PRP group and autologous nerve transplantation group had similar benefits in reducing atrophy of the target organ. In the same nerve defect model, Abbasipour dalivand and other scholars used silica gel tube to bridge the defect in rats, and evaluated the effect of PRP on nerve regeneration. At 4, 6, 8 weeks after operation, PRP improved nerve regeneration significantly. Abbasipour-Dalivand et al. (2015) studied the effect of PRP and SCs (differentiated from rabbit mesenchymal stem cells) on nerve regeneration in a nerve defect model using tissue engineering neurology. The control group was fibrin combined with SCs added into the tissue engineering nerve. According to their results, the number of regenerated nerve fibers and the thickness of myelin sheath in the tissue engineering group with PRP and SCs were higher than those in the control group. Also, the compound muscle action potential and nerve conduction were better than those in the control group. As such, the authors concluded that PRP gel is a good medium for SCs and can effectively promote nerve regeneration. Ye et al. (2012) Ikumi made PRF into membrane and wrapped it in the anastomotic site of rabbit sciatic nerve model. Although he did not find significant differences in electrophysiological recovery and muscle wet weight ratio recovery between the experimental group and the control group, the SC activation degree and the number, as well as diameter of axon regeneration in the experimental group were significantly higher than those in the control group (Ikumi et al., 2018). Application of low-frequency shock wave combined with local injection of PRP in the treatment of rabbit sciatic nerve crush injury has also been effective (Zhu et al., 2020).

In recent years, renewed type of PRP and combination with other materials, such as stem cell or tissue engineering neural conduit, are utilized in peripheral nerve regeneration and recent process were summarized in Table 4.

**TABLE 4:** Summary of some recent process of PRP on peripheral nerve regeneration.

Type of study	Model	Intervention	Outcome	Ref
*In vivo*	cavernous nerve injury in rats	intracavernosal (IC) injection of chitosan activated platelet rich plasma (cPRP)	This simultaneous accelerated the regeneration of myelinated axons of the cavernous nerve, reduced apoptosis, and enhanced the proliferation of the corporal smooth muscle cells at an earlier stage	Wu et al. (2022)
*In vivo*	Bilateral cavernous nerve injury in rats	Probed samples using a cytokine antibody array and performed ELISA	This study provides evidence for the role of CXCL5 and CXCR2 as mediators of PRP effects in the preservation of EF after CN injury	Wu et al. (2021)
*In vivo*	facial nerve regeneration in rabbits	a titanium-prepared platelet-rich fibrin membrane was wrapped in a tube around the damaged area	local T-PRF membrane application positively contributes to observable improvement in facial movements following healing after facial nerve injury. Electrophysiologically, the warning threshold findings were determined to recover earlier. Longer followup studies with standard methods, in which surgical and treatment protocols can be refined, can be of benefit in clarifying the effects of PRF on nerve healing	Şentürk et al. (2020)
*In vivo*	Facial nerve regeneration in rats	The injured nerves were covered with spongostan impregnated with a solution of CHT with PRP (1:1)	Chitosan gel has a positive efect on nerve healing and applying it along with PRP can enhance the effect of chitosan	Şahin et al. (2021)
*In vitro* and *in vivo*	SCs proliferation *in vitro* and Sciatic nerve defect in rats	Sophisticated polycaprolactone/gelatin nanofibrous nerve guided conduit containing platelet-rich plasma	The fabricated nerve guide conduit exhibited promising physicochemical and biological activates favorable for PNI treatment	Samadian et al. (2020)
*In vivo*	Sciatic nerve defect in rats	PRFr (0.1 ml) and adipose derived stem cells (10^6^ cells/0.1 ml) were injected into the defect site	This injection approach may provide a successfully employed technique to target sciatic nerve defects *in vivo*, and the combined strategy of ADSCs with PRFr appears to have a superior effect on nerve repair	Chuang et al. (2020)
*In vivo*	Sciatic nerves in Rats	PRP infiltration in defect site	PRP had a significant effect (*p* < 0.05) on the sciatic nerve repair when compared with the control group	Kokkalas et al. (2020)
*In vivo*	Facial nerve injury in rats	PRF infiltration conbined with topical tacrolimus in defect site	compared to PRF alone, PRF with topical tacrolimus application led to the best regeneration result after a FN crush injury	Mourad et al. (2021)

### The Clinical Application of Platelet Rich Plasma in Promoting the Peripheral Nerve Regeneration

Peripheral nerve injury is a common clinical nervous system disease with a substantial impact on the health and quality of life of patients. The condition can be caused by several factors. Common causes include nerve continuity interruption due to trauma, and peripheral nerve compression damage caused by anatomical structure variation of specific parts of nerve (also known as peripheral nerve entrapment syndrome), such as cubital tunnel syndrome, carpal tunnel syndrome, dorsal interosseous nerve compression syndrome (supinator syndrome), and interosseous palmar nerve entrapment syndrome (Pronator teres syndrome). There are also peripheral nerve demyelinating lesions caused by immune system diseases, such as Guillain Barre syndrome. Peripheral nerve damage can also be caused by diabetes. In recent years, clinical studies have revealed the therapeutic effect of PRP on peripheral nerve injury. More research has focused on the treatment of carpal tunnel syndrome.

Shen et al. (2019) conducted a prospective, randomized, single-blind, head-to-head clinical trial involving 52 patients with unilateral moderate carpal tunnel syndrome. The study compared the effects of ultrasound-guided local injection of PRP and 5% glucose on carpal tunnel nerve recovery. Patients who received a single dose of PRP had better postoperative (Boston carpal tunnel questionnaire, BCTQ) and neuroelectrophysiological recovery than those who received glucose injection. Local injection of glucocorticoid is one of the traditional conservative methods for treating patients with carpal tunnel syndrome. A study compared the efficacy of local glucocorticoid injection and PRP injection in the treatment of patients with mild carpal tunnel syndrome, and found that the clinical effect of PRP local injection was superior to hormone treatment (Uzun et al., 2017). In a prospective randomized controlled study, Catapano et al. (2020). Compared the hand holding strength, postoperative rest days, Visual analog score (VAS), and BCTQ between two groups of patients. According to their results, the use of PRP as an adjuvant treatment of carpal tunnel syndrome was superior and effective in improving the prognosis of patients. Another prospective (Wu et al., 2017), randomized, single-blind, controlled trial confirmed that patients with carpal tunnel syndrome who received PRP exhibited a better prognosis. Collectively, these studies indicate that PRP plays an active role in the repair of peripheral nerve injury.

In recent years, many exciting case reports on other types of peripheral nerve injury have emerged. García de Cortázar et al. (2018) reported a 28-year-old female patient with finger nerve entrapment, who suffered from loss of index finger sensation and nerve pain for a long time. Therefore, neurolysis and PRP injection were performed during her operation. In the early postoperative period, the neuralgia of the patient was significantly relieved, and the finger nerve showed signs of recovery. Besides, there was no adverse reaction from the PRP application. These findings further suggest that PRP can play a positive role in alleviating neuralgia and promoting the recovery of nerve function. Studies (Sánchez et al., 2014) have also shown that local ultrasound injection of PRP can effectively cure patients with post-traumatic peroneal nerve palsy. Kuffler (Kuffler et al., 2011) successfully treated a 12 cm ulnar nerve defect, which had been injured for 3.25 years with PRF filled fibrin nerve conduit, and successfully restored the normal lifestyle of a patient suffering from ulnar nerve function and neuralgia. Additionally, Kuffler (Kuffler, 2013) found that PRF could promote nerve regeneration and functional recovery, and effectively alleviate the symptoms of neuralgia. These results further clarified the role and mechanism of PRP in relieving neuralgia. Collectively, these cases further reveal the enormous therapeutic potential of PRP. In addition to the common peripheral nerve injury of limbs, the incidence of nerve injury in maxillofacial surgery is relatively common. Sagittal osteotomy of mandibular ramus is a common operation in maxillofacial surgery. Tabrizi et al. (2018) carried out a double-blind randomized controlled clinical study to determine whether PRP can promote alveolar nerve recovery after sagittal osteotomy. According to their results, PRF was effective in the treatment of injured alveolar nerve. The clinical application of PRP on peripheral nerve regeneration was summerized in Table 5.

**TABLE 5:** Summary of clinical application of PRP on peripheral nerve regeneration.

Type of study	Clinical case	Intervention	Outcome	References
Case report *n* = 52	unilateral moderate carpal tunnel syndrome	ultrasound-guided local injection of PRP or 5% glucose	Patients who received a single dose of PRP had better postoperative (Boston carpal tunnel questionnaire, BCTQ) and neuroelectrophysiological recovery than those who received glucose injection	Shen et al. (2019)
Case report *n* = 40	Mild carpal tunnel syndrome	Local glucocorticoid injection or PRP injection	Clinical effect of PRP local injection was superior to hormone treatment	Uzun et al. (2017)
Mata analysis *n* = 191	carpal tunnel syndrome	PRP injection	the use of PRP as an adjuvant treatment of carpal tunnel syndrome was superior and effective in improving the prognosis of patients	Catapano et al. (2020)
Case report *n* = 60	carpal tunnel syndrome	Injected with one dose of 3 ml of PRP using ultrasound guidance and the control group received a night splint	The PRP group exhibited a significant reduction in the VAS score, BCTQ score, and CSA of MN compared to the those of control group 6 months post-treatment	Wu et al. (2017)
Case report n = 1	Radial nerve injury	Four months after the trauma, serial intraneural infiltrations of PRP were conducted using ultrasound guidance	Fourteen months after the injury and 11 months after the first PRP injection, functional recovery was achieved. The EMG showed a complete reinnervation of the musculature of the radial nerve dependent	García de Cortázar et al. (2018)
Case report *n* = 1	Peroneal nerve palsy in traumatic knee dislocations	Eleven months after the trauma with severe axonotmesis, serial intraneural infiltrations of PRGF were started using ultrasound guidance	Plasma rich in growth factors (PRGF) infiltrations enhanced healing process of peroneal nerve palsy with drop foot	Sánchez et al. (2014)
Case report *n* = 1	peripheral nerve gaps	A 12-cm-long nerve gap was bridged with a collagen tube filled with autologous platelet-rich fibrin	The conduit filled with platelet-rich fibrin can induce limited, but appropriate, sensory and motor recovery across a 12-cm nerve gap repaired 3.25 years post trauma, without sacrificing a sensory nerve, can reduce existing excruciating neuropathic pain to tolerable, and allow avoidance of an indicated upper-extremity amputation	Kuffler et al. (2011)
Case report *n* = 1	Peripheral neuropathic pain	A 12-cm-long nerve gap was bridged with a collagen tube filled with autologous platelet-rich fibrin	PRF could promote nerve regeneration and functional recovery, and effectively alleviate the symptoms of neuralgia	Kuffler, (2013)
Case report *n* = 1	A zone 4–5 ulnar nerve laceration	Damaged tissue was removed and bridged with 22-cm length of sural nerve which was surrounded with collagen tube combined with PRP.	After 2 years follow-up, the patient got a satisfactory recovery of the function of ulnar nerve	Foy et al. (2021)
Case report *n* = 21	Neurosensory disturbance (NSD) after sagittal split osteotomy (SSO) surgery	PRF was applied after the osteotomy and before fixation on one osteotomy side. The other osteotomy side served as the control group	PRF may enhance the recovery of paresthesia following SSO. All subjective tests showed enhanced recovery of NSD.	Tabrizi et al. (2018)

There is a controversy regarding the whether PRP is beneficial or not in clinic. In my opinion, the method standardization is not the only aspect that caused the conflicting results. PRP is with complex composition, and has been widely studied for its regenerative potential, which is ascribed to its capability to secrete growth factors, cytokines, and ECM, thereby promoting migration, proliferation, stabilization, and differentiation of endothelial, fibroblast, and stem cells. Due to different preparation methods, the components of PRP are different, among which the number of leukocytes is an important criterion (Huang et al., 2017). According to the different concentration of leukocytes in the PRP, PRP can be classified into Leukocyte poor platele-rich Plasma (P-PRP) and Leukocyte Rich PRP (L-PRP). PRP with different leukocyte content has different effects on cell regeneration and tissue healing. The leukocytes in L-PRP will increase the level of pro-inflammatory cytokine, including IL-1and TNF-α, further induced inflammation in normal and injured tissue. Yin et al. compared the effect of L-PRP and P-PRP on extracellular matrix formation and the NF-κB signaling pathway in human articular chondrocytes. This report indicated that suggested that leukocytes in L-PRP activated the NF-κB signaling pathway *via* the delivery of IL-1 and TNF-α to counter the beneficial effects of growth factors on extracellular matrix formation in HACs. P-PRP may be more suitable for the treatment of osteoarthritis (Yin et al., 2017). Zhou et al. compared the effect of L-PRP and P-PRP on cartilage regeneration in a cartilage defects model in rabbits and found that leukocytes in L-PRP might activate NF-κB pathway *via* the delivery of IL-1 beta and TNF-α to induce harmful effects on cartilage regeneration and result in the inferior effects of L-PRP compared with P-PRP. P-PRP may be more suitable than L-PRP for the treatment of cartilage defects (Xu et al., 2017). From a physiopathological point of view, in some situation, excessive inflammatory response may lead to hyperplasia of fibrillar connective tissue around the site of nerve injury. This hyperplasia could cause nerve compression or gap. However, few report support this side effect of L-PRP on nerve regeneration. On the contrary, many reports have indicate the positive effect of the appropriate inflammatory response induced by L-PRP or L-PRF on peripheral nerve regeneration (Wang et al., 2020).

Beside, PRP is activated to release growth factors by using stimulants such as thrombin, calcium chloride, and collagen. However, the exact concentration and procedure for PRP activation is not well established (Sánchez et al., 2017b). The controversy around PRP in clinic is complex. It relates to lots of factors such as components, indications, activation condition, and so on. In order to establish the role of PRP in clinic fully, extensive clinical studies are required to optimize the therapeutic protocol and hence the efficacy and safety.

To sum up, studies on the application of PRP in the treatment of peripheral nerve injury have increased substantially in the past 10 years. It is noteworthy that most of the studies have revealed that patients with peripheral nerve injury receiving PRP treatment have a rapid recovery. However, some studies have reported conflicting results on whether PRP can promote peripheral nerve regeneration. For example, Raeissadat et al. (2018) conducted a clinical randomized controlled trial on the treatment of carpal tunnel syndrome using PRP, and found no significant difference in the outcome between the PRP group and the control group. It should be noted that in the clinical application of PRP, the production methods (including centrifugal time, relative centrifugal force, etc.) have not been standardized, and thus, PRP products produced at present do not have uniform quality control. Therefore, uniformity in PRP production process and better-quality control in the future is likely to expand its application in other fields and further realize its potential in clinical treatment.

### Prospect of Clinical Application of Platelet Rich Plasma

PRP is widely used to promote tissue repair and regeneration because of its abundant cytokines. Recently, a study evaluated the current status of PRP research worldwide using bibliometrics. A total of 8,499 studies on PRP were retrieved, and the number of studies and citations is increasing yearly. Most studies have focused on basic research of growth factor function, bone regeneration, cartilage and osteoarthritis-related fields, platelet function research, and stem cell-related research fields. Regarding clinical application, most studies (Xing, 2020) have mainly focused on orthopedics, especially cartilage regeneration and osteoarthritis. In the aspect of peripheral nerve injury, the number of related literature is increasing yearly. Although most studies are still based on animal experiments and *in vitro* studies, the clinical application of PRP in the treatment of peripheral nerve injury is also increasing. There is substantial evidence of the effectiveness of PRP in promoting nerve regeneration. Studies focusing on combining PRP, stem cell into tissue engineering nerve in the treatment of nerve defects are also on the increase and the technology is expected to replace nerve transplantation.

## References

[B1] Abbasipour-Dalivand S. Mohammadi R. Mohammadi V. (2015). Effects of Local Administration of Platelet Rich Plasma on Functional Recovery after Bridging Sciatic Nerve Defect Using Silicone Rubber Chamber; an Experimental Study. Bull. Emerg. Trauma 3 (1), 1–7. 27162893 PMC4771280

[B2] Caillaud M. Richard L. Vallat J. M. Desmoulière A. Billet F. (2019). Peripheral Nerve Regeneration and Intraneural Revascularization. Neural Regen. Res. 14 (1), 24–33. 10.4103/1673-5374.243699 30531065 PMC6263011

[B3] Castro M. V. d. Silva M. V. R. d. Chiarotto G. B. Volpe B. B. Santana M. H. Malheiros Luzo Â. C. (2019). Reflex Arc Recovery after Spinal Cord Dorsal Root Repair with Platelet Rich Plasma (PRP). Brain Res. Bull. 152, 212–224. 10.1016/j.brainresbull.2019.07.024 31351157

[B4] Catapano M. Catapano J. Borschel G. Alavinia S. M. Robinson L. R. Mittal N. (2020). Effectiveness of Platelet-Rich Plasma Injections for Nonsurgical Management of Carpal Tunnel Syndrome: A Systematic Review and Meta-Analysis of Randomized Controlled Trials. Arch. Phys. Med. Rehabil. 101 (5), 897–906. 10.1016/j.apmr.2019.10.193 31821797

[B5] Cattin A.-L. Burden J. J. Van Emmenis L. Mackenzie F. E. Hoving J. J. A. Garcia Calavia N. (2015). Macrophage-Induced Blood Vessels Guide Schwann Cell-Mediated Regeneration of Peripheral Nerves. Cell 162 (5), 1127–1139. 10.1016/j.cell.2015.07.021 26279190 PMC4553238

[B6] Chuang M. H. Ho L. H. Kuo T. F. Sheu S. Y. Liu Y. H. Lin P. C. (2020). Regenerative Potential of Platelet-Rich Fibrin Releasate Combined with Adipose Tissue-Derived Stem Cells in a Rat Sciatic Nerve Injury Model. Cell Transpl. 29, 963689720919438. 10.1177/0963689720919438 PMC7586258 32538130

[B7] Delavary B. M. VAN der Veer W. M. VAN Egmond M. Niessen F. B. Beelen R. H. J. (2011). Macrophages in Skin Injury and Repair. Immunobiology 216 (7), 753–762. 10.1016/j.imbio.2011.01.001 21281986

[B8] Dohan D. M. Choukroun J. Diss A. Dohan S. L. Dohan A. J. J. Mouhyi J. (2006). Platelet-rich Fibrin (PRF): A Second-Generation Platelet Concentrate. Part II: Platelet-Related Biologic Features. Oral Surg. Oral Med. Oral Pathol. Oral Radiol. Endodontology 101 (3), e45–e50. 10.1016/j.tripleo.2005.07.009 16504850

[B9] Dun X. P. Parkinson D. B. (2017). Role of Netrin-1 Signaling in Nerve Regeneration. Int. J. Mol. Sci. 18 (3). 10.3390/ijms18030491 PMC5372507 28245592

[B10] Duymus T. M. Mutlu S. Dernek B. Komur B. Aydogmus S. Kesiktas F. N. (2017). Choice of Intra-articular Injection in Treatment of Knee Osteoarthritis: Platelet-Rich Plasma, Hyaluronic Acid or Ozone Options. Knee Surg. Sports Traumatol. Arthrosc. 25 (2), 485–492. 10.1007/s00167-016-4110-5 27056686

[B11] Etulain J. (2018). Platelets in Wound Healing and Regenerative Medicine. Platelets. 29 (6), 556–568. 10.1080/09537104.2018.1430357 29442539

[B12] Fawcett J. W. Keynes R. J. (1990). Peripheral Nerve Regeneration. Annu. Rev. Neurosci. 13, 43–60. 10.1146/annurev.ne.13.030190.000355 2183684

[B13] Firat C. Aytekin A. H. Durak M. A. Geyik Y. Erbatur S. Dogan M. (2016). Comparison of the Effects of PRP and Hyaluronic Acid in Promoting Peripheral Nerve Regeneration an Experimental Study with Vascular Conduit Model in Rats'. Ann. Ital. Chir 87, 362–374. 27680608

[B14] Fowler J. R. Lavasani M. Huard J. Goitz R. J. (2015). Biologic Strategies to Improve Nerve Regeneration after Peripheral Nerve Repair. J. Reconstr. Microsurg 31 (4), 243–248. 10.1055/s-0034-1394091 25503421

[B15] Foy C. A. Micheo W. F. Kuffler D. P. (2021). Functional Recovery Following Repair of Long Nerve Gaps in Senior Patient 2.6 Years Posttrauma. Plast. Reconstr. Surg. Glob. Open 9 (9), e3831. 10.1097/gox.0000000000003831 34584828 PMC8460218

[B16] García de Cortázar U. Padilla S. Lobato E. Delgado D. Sánchez M. (2018). Intraneural Platelet-Rich Plasma Injections for the Treatment of Radial Nerve Section: A Case Report. J. Clin. Med. 7 (2). 10.3390/jcm7020013 PMC5852429 29382110

[B17] García-Conca V. Abad-Collado M. Hueso-Abancens J. R. Mengual-Verdú E. Piñero D. P. Aguirre-Balsalobre F. (2019). Efficacy and Safety of Treatment of Hyposecretory Dry Eye with Platelet-Rich Plasma. Acta Ophthalmol. 97 (2), e170–e8. 10.1111/aos.13907 30450721

[B18] Ghanaati S. Booms P. Orlowska A. Kubesch A. Lorenz J. Rutkowski J. (2014). Advanced Platelet-Rich Fibrin: A New Concept for Cell-Based Tissue Engineering by Means of Inflammatory Cells. J. Oral Implantol. 40 (6), 679–689. 10.1563/aaid-joi-d-14-00138 24945603

[B19] Hillenbrand M. Holzbach T. Matiasek K. Schlegel J. Giunta R. E. (2015). Vascular Endothelial Growth Factor Gene Therapy Improves Nerve Regeneration in a Model of Obstetric Brachial Plexus Palsy. Neurol. Res. 37 (3), 197–203. 10.1179/1743132814y.0000000441 25213596

[B20] Huang M.-L. Zhai Z. Chen Z.-X. Yang X.-N. Qi Z.-L. (2020). Platelet-rich Fibrin Membrane Nerve Guidance Conduit: a Potentially Promising Method for Peripheral Nerve Injuries. Chin. Med. J. (Engl). 133 (8), 999–1001. 10.1097/cm9.0000000000000726 32187061 PMC7176438

[B21] Huang Y. Bornstein M. M. Lambrichts I. Yu H.-Y. Politis C. Jacobs R. (2017). Platelet-rich Plasma for Regeneration of Neural Feedback Pathways Around Dental Implants: a Concise Review and Outlook on Future Possibilities. Int. J. Oral Sci. 9 (1), 1–9. 10.1038/ijos.2017.1 28282030 PMC5379164

[B22] Ikumi A. Hara Y. Yoshioka T. Kanamori A. Yamazaki M. (2018). Effect of Local Administration of Platelet-Rich Plasma (PRP) on Peripheral Nerve Regeneration: An Experimental Study in the Rabbit Model. Microsurgery. 38 (3), 300–309. 10.1002/micr.30263 29094404

[B23] Jessen K. R. Mirsky R. (2016). The Repair Schwann Cell and its Function in Regenerating Nerves. J. Physiol. 594 (13), 3521–3531. 10.1113/jp270874 26864683 PMC4929314

[B24] Kaminski R. Maksymowicz-Wleklik M. Kulinski K. Kozar-Kaminska K. Dabrowska-Thing A. Pomianowski S. (2019). Short-Term Outcomes of Percutaneous Trephination with a Platelet Rich Plasma Intrameniscal Injection for the Repair of Degenerative Meniscal Lesions. A Prospective, Randomized, Double-Blind, Parallel-Group, Placebo-Controlled Study. Int. J. Mol. Sci. 20 (4). 10.3390/ijms20040856 PMC6412887 30781461

[B25] Kokkalas N. Kokotis P. Diamantopoulou K. Galanos A. Lelovas P. Papachristou D. J. (2020). Platelet-rich Plasma and Mesenchymal Stem Cells Local Infiltration Promote Functional Recovery and Histological Repair of Experimentally Transected Sciatic Nerves in Rats. Cureus 12 (5), e8262. 10.7759/cureus.8262 32596080 PMC7313431

[B26] Kroner A. Greenhalgh A. D. Zarruk J. G. Passos dos Santos R. Gaestel M. David S. (2014). TNF and Increased Intracellular Iron Alter Macrophage Polarization to a Detrimental M1 Phenotype in the Injured Spinal Cord. Neuron 83 (5), 1098–1116. 10.1016/j.neuron.2014.07.027 25132469

[B27] Kuffler D. P. Foy C. (2020). Restoration of Neurological Function Following Peripheral Nerve Trauma. Int. J. Mol. Sci. 21 (5). 10.3390/ijms21051808 PMC7084579 32155716

[B28] Kuffler D. P. (2013). Platelet-Rich Plasma and the Elimination of Neuropathic Pain. Mol. Neurobiol. 48 (2), 315–332. 10.1007/s12035-013-8494-7 23832571

[B29] Kuffler D. P. Reyes O. Sosa I. J. Santiago-Figueroa J. (2011). Neurological Recovery across a 12-Cm-Long Ulnar Nerve Gap Repaired 3.25 Years Post Trauma: Case Report. Neurosurgery 69 (6), E1321–E1326. 10.1227/neu.0b013e31822a9fd2 21712738

[B30] Lana J. F. Huber S. C. Purita J. Tambeli C. H. Santos G. S. Paulus C. (2019). Leukocyte-rich PRP versus Leukocyte-Poor PRP - the Role of Monocyte/macrophage Function in the Healing cascade. J. Clin. Orthopaedics Trauma 10 (Suppl. 1), S7–S12. 10.1016/j.jcot.2019.05.008 PMC6823808 31700202

[B31] Lisi C. Perotti C. Scudeller L. Sammarchi L. Dametti F. Musella V. (2018). Treatment of Knee Osteoarthritis: Platelet-Derived Growth Factors vs. Hyaluronic Acid. A Randomized Controlled Trial. Clin. Rehabil. 32 (3), 330–339. 10.1177/0269215517724193 28783969

[B32] Lu J. Yan X. Sun X. Shen X. Yin H. Wang C. (2019). Synergistic Effects of Dual-Presenting VEGF- and BDNF-Mimetic Peptide Epitopes from Self-Assembling Peptide Hydrogels on Peripheral Nerve Regeneration. Nanoscale 11 (42), 19943–19958. 10.1039/c9nr04521j 31602446

[B33] Merolla G. Dellabiancia F. Ricci A. Mussoni M. P. Nucci S. Zanoli G. (2017). Arthroscopic Debridement versus Platelet-Rich Plasma Injection: A Prospective, Randomized, Comparative Study of Chronic Lateral Epicondylitis with a Nearly 2-Year Follow-Up. Arthrosc. J. Arthroscopic Relat. Surg. 33 (7), 1320–1329. 10.1016/j.arthro.2017.02.009 28433443

[B34] Min Q. Parkinson D. B. Dun X. P. (2021). Migrating Schwann Cells Direct Axon Regeneration within the Peripheral Nerve Bridge. Glia. 69 (2), 235–254. 10.1002/glia.23892 32697392

[B35] Miron R. J. Fujioka-Kobayashi M. Hernandez M. Kandalam U. Zhang Y. Ghanaati S. (2017). Injectable Platelet Rich Fibrin (I-PRF): Opportunities in Regenerative Dentistry? Clin. Oral Invest. 21 (8), 2619–2627. 10.1007/s00784-017-2063-9 28154995

[B36] Mishra A. K. Skrepnik N. V. Edwards S. G. Jones G. L. Sampson S. Vermillion D. A. (2014). Efficacy of Platelet-Rich Plasma for Chronic Tennis Elbow. Am. J. Sports Med. 42 (2), 463–471. 10.1177/0363546513494359 23825183

[B37] Mohamadi S. Norooznezhad A. H. Mostafaei S. Nikbakht M. Nassiri S. safar H. (2019). A Randomized Controlled Trial of Effectiveness of Platelet-Rich Plasma Gel and Regular Dressing on Wound Healing Time in Pilonidal Sinus Surgery: Role of Different Affecting Factors. Biomed. J. 42 (6), 403–410. 10.1016/j.bj.2019.05.002 31948604 PMC6963006

[B38] Mourad S. I. Al-Dubai S. A. Elsayed S. A. El-Zehary R. R. (2021). Efficacy of Platelet-Rich Fibrin and Tacrolimus on Facial Nerve Regeneration: an Animal Study. Int. J. Oral Maxillofac. Surg. 51, 279-287. 10.1016/j.ijom.2021.05.016 34090756

[B39] Nishio H. Saita Y. Kobayashi Y. Takaku T. Fukusato S. Uchino S. (2020). Platelet-rich Plasma Promotes Recruitment of Macrophages in the Process of Tendon Healing. Regenerative Ther. 14, 262–270. 10.1016/j.reth.2020.03.009 PMC7232040 32455156

[B40] Painter M. W. Brosius Lutz A. Cheng Y.-C. Latremoliere A. Duong K. Miller C. M. (2014). Diminished Schwann Cell Repair Responses Underlie Age-Associated Impaired Axonal Regeneration. Neuron 83 (2), 331–343. 10.1016/j.neuron.2014.06.016 25033179 PMC4106408

[B41] Parrinello S. Napoli I. Ribeiro S. Digby P. W. Fedorova M. Parkinson D. B. (2010). EphB Signaling Directs Peripheral Nerve Regeneration through Sox2-dependent Schwann Cell Sorting. Cell. 143 (1), 145–155. 10.1016/j.cell.2010.08.039 20869108 PMC3826531

[B42] Peerbooms J. C. Lodder P. Den Oudsten B. L. Doorgeest K. Schuller H. M. Gosens T. (2019). Positive Effect of Platelet-Rich Plasma on Pain in Plantar Fasciitis: A Double-Blind Multicenter Randomized Controlled Trial. Am. J. Sports Med. 47 (13), 3238–3246. 10.1177/0363546519877181 31603721

[B43] Pelletier J. Roudier E. Abraham P. Fromy B. Saumet J. L. Birot O. (2015). VEGF-A Promotes Both Pro-angiogenic and Neurotrophic Capacities for Nerve Recovery after Compressive Neuropathy in Rats. Mol. Neurobiol. 51 (1), 240–251. 10.1007/s12035-014-8754-1 24865514

[B44] Pereira C. T. Paxton Z. J. Li A. I. (2020). Involvement of PDGF-BB and IGF-1 in Activation of Human Schwann Cells by Platelet-Rich Plasma. Plast. Reconstr. Surg. 146 (6), 825e–827e. 10.1097/prs.0000000000007406 33234996

[B45] Pereira Lopes F. R. Lisboa B. C. G. Frattini F. Almeida F. M. Tomaz M. A. Matsumoto P. K. (2011). Enhancement of Sciatic Nerve Regeneration after Vascular Endothelial Growth Factor (VEGF) Gene Therapy. Neuropathol. Appl. Neurobiol. 37 (6), 600–612. 10.1111/j.1365-2990.2011.01159.x 21208251

[B46] Qin J. Wang L. Sun Y. Sun X. Wen C. Shahmoradi M. (2016). Concentrated Growth Factor Increases Schwann Cell Proliferation and Neurotrophic Factor Secretion and Promotes Functional Nerve Recovery *In Vivo* . Int. J. Mol. Med. 37 (2), 493–500. 10.3892/ijmm.2015.2438 26709397

[B47] Qin J. Wang L. Zheng L. Zhou X. Zhang Y. Yang T. (2016). Concentrated Growth Factor Promotes Schwann Cell Migration Partly through the Integrin β1-mediated Activation of the Focal Adhesion Kinase Pathway. Int. J. Mol. Med. 37 (5), 1363–1370. 10.3892/ijmm.2016.2520 26986804

[B48] Qu W. R. Zhu Z. Liu J. Song D. B. Tian H. Chen B. P. (2021). Interaction between Schwann Cells and Other Cells during Repair of Peripheral Nerve Injury. Neural Regen. Res. 16 (1), 93–98. 10.4103/1673-5374.286956 32788452 PMC7818858

[B49] Raeissadat S. A. Karimzadeh A. Hashemi M. Bagherzadeh L. (2018). Safety and Efficacy of Platelet-Rich Plasma in Treatment of Carpal Tunnel Syndrome; a Randomized Controlled Trial. BMC Musculoskelet. Disord. 19 (1), 49. 10.1186/s12891-018-1963-4 29433485 PMC5810049

[B50] Roth F. Fernandes M. Valente S. G. Santos J. B. G. Furukawa R. B. Fernandes C. H. (2017). Platelet-Rich Fibrin Conduits as an Alternative to Nerve Autografts for Peripheral Nerve Repair. J. Reconstr. Microsurg 33 (8), 549–556. 10.1055/s-0037-1603355 28561191

[B51] Şahin M. M. Cayonu M. Dinc S. K. Ozkocer E. Ilhan M. Uzunoğlu E. (2021). Effects of Chitosan and Platelet-Rich Plasma on Facial Nerve Regeneration in an Animal Model. Eur. Arch. Otorhinolaryngol. 279, 987-994. 10.1007/s00405-021-06859-6 33956207

[B52] Samadian H. Ehterami A. Sarrafzadeh A. Khastar H. Nikbakht M. Rezaei A. (2020). Sophisticated Polycaprolactone/gelatin Nanofibrous Nerve Guided Conduit Containing Platelet-Rich Plasma and Citicoline for Peripheral Nerve Regeneration: *In Vitro* and *In Vivo* Study. Int. J. Biol. Macromolecules 150, 380–388. 10.1016/j.ijbiomac.2020.02.102 32057876

[B53] Sánchez M. Garate A. Delgado D. Padilla S. (2017). Correction: Platelet-Rich Plasma, an Adjuvant Biological Therapy to Assist Peripheral Nerve Repair. Neural Regen. Res. 12 (1), 338–352. 10.4103/1673-5374.202914 28250739 PMC5319232

[B54] Sánchez M. Anitua E. Delgado D. Sanchez P. Prado R. Orive G. (2017). Platelet-rich Plasma, a Source of Autologous Growth Factors and Biomimetic Scaffold for Peripheral Nerve Regeneration. Expert Opin. Biol. Ther. 17 (2), 197–212. 10.1080/14712598.2017.1259409 27845852

[B55] Sánchez M. Yoshioka T. Ortega M. Delgado D. Anitua E. (2014). Ultrasound-guided Platelet-Rich Plasma Injections for the Treatment of Common Peroneal Nerve Palsy Associated with Multiple Ligament Injuries of the Knee. Knee Surg. Sports Traumatol. Arthrosc. 22 (5), 1084–1089. 10.1007/s00167-013-2479-y 23519544

[B56] Şentürk F. Bahadır O. Aktaş O. Bıyık A. F. Ercan E. (2020). Effects of Titanium Prepared Platelet Rich Fibrin on Facial Nerve Regeneration: an Experimental Study. Braz. J. Otorhinolaryngol. 10.1016/j.bjorl.2020.11.014 PMC9615514 33441277

[B57] Shen Y. P. Li T. Y. Chou Y. C. Ho T. Y. Ke M. J. Chen L. C. (2019). Comparison of Perineural Platelet‐rich Plasma and Dextrose Injections for Moderate Carpal Tunnel Syndrome: A Prospective Randomized, Single‐blind, Head‐to‐head Comparative Trial. J. Tissue Eng. Regen. Med. 13 (11), 2009–2017. 10.1002/term.2950 31368191

[B58] Stolle M. Schulze J. Roemer A. Lenarz T. Durisin M. Warnecke A. (2018). Human Plasma Rich in Growth Factors Improves Survival and Neurite Outgrowth of Spiral Ganglion Neurons *In Vitro* . Tissue Eng. Part. A. 24 (5-6), 493–501. 10.1089/ten.TEA.2017.0120 28610547

[B59] Stratton J. A. Holmes A. Rosin N. L. Sinha S. Vohra M. Burma N. E. (2018). Macrophages Regulate Schwann Cell Maturation after Nerve Injury. Cell Rep. 24 (10), 2561–2572. 10.1016/j.celrep.2018.08.004 30184491

[B60] Stratton J. A. Shah P. T. Kumar R. Stykel M. G. Shapira Y. Grochmal J. (2016). The Immunomodulatory Properties of Adult Skin-Derived Precursor Schwann Cells: Implications for Peripheral Nerve Injury Therapy. Eur. J. Neurosci. 43 (3), 365–375. 10.1111/ejn.13006 26121489

[B61] Stratton J. Shah P. (2016). Macrophage Polarization in Nerve Injury: Do Schwann Cells Play a Role? Neural Regen. Res. 11 (1), 53–57. 10.4103/1673-5374.175042 26981078 PMC4774224

[B62] Tabrizi R. Pourdanesh F. Jafari S. Behnia P. (2018). Can Platelet-Rich Fibrin Accelerate Neurosensory Recovery Following Sagittal Split Osteotomy? A Double-Blind, Split-Mouth, Randomized Clinical Trial. Int. J. Oral Maxillofacial Surg. 47 (8), 1011–1014. 10.1016/j.ijom.2018.04.010 30954205

[B63] Takeuchi M. Kamei N. Shinomiya R. Sunagawa T. Suzuki O. Kamoda H. (2012). Human Platelet-Rich Plasma Promotes Axon Growth in Brain-Spinal Cord Coculture. Neuroreport. 23 (12), 712–716. 10.1097/wnr.0b013e3283567196 22750774

[B64] Toews A. D. Barrett C. Morell P. (1998). Monocyte Chemoattractant Protein 1 Is Responsible for Macrophage Recruitment Following Injury to Sciatic Nerve. J. Neurosci. Res. 53 (2), 260–267. 10.1002/(sici)1097-4547(19980715)53:2<260::aid-jnr15>3.0.co;2-a 9671983

[B65] Torigoe K. Tanaka H.-F. Takahashi A. Awaya A. Hashimoto K. (1996). Basic Behavior of Migratory Schwann Cells in Peripheral Nerve Regeneration. Exp. Neurol. 137 (2), 301–308. 10.1006/exnr.1996.0030 8635545

[B66] Ucak Turer O. Ozcan M. Alkaya B. Surmeli S. Seydaoglu G. Haytac M. C. (2020). Clinical Evaluation of Injectable Platelet‐rich Fibrin with Connective Tissue Graft for the Treatment of Deep Gingival Recession Defects: A Controlled Randomized Clinical Trial. J. Clin. Periodontol. 47 (1), 72–80. 10.1111/jcpe.13193 31518440

[B67] Uzun H. Bitik O. Uzun Ö. Ersoy U. S. Aktaş E. (2017). Platelet-rich Plasma versus Corticosteroid Injections for Carpal Tunnel Syndrome. J. Plast. Surg. Hand Surg. 51 (5), 301–305. 10.1080/2000656x.2016.1260025 27921443

[B68] Wang Z. Mudalal M. Sun Y. Liu Y. Wang J. Wang Y. (2020). The Effects of Leukocyte-Platelet Rich Fibrin (L-PRF) on Suppression of the Expressions of the Pro-inflammatory Cytokines, and Proliferation of Schwann Cell, and Neurotrophic Factors. Sci. Rep. 10 (1), 2421. 10.1038/s41598-020-59319-2 32051476 PMC7016122

[B69] Wu Y.-N. Liao C.-H. Chen K.-C. Chiang H.-S. (2021). CXCL5 Cytokine Is a Major Factor in Platelet-Rich Plasma's Preservation of Erectile Function in Rats after Bilateral Cavernous Nerve Injury. J. Sex. Med. 18 (4), 698–710. 10.1016/j.jsxm.2020.12.016 33741291

[B70] Wu Y.-T. Ho T. Y. Chou Y.-C. Ke M.-J. Li T.-Y. Huang G.-S. (2017). Six-month Efficacy of Platelet-Rich Plasma for Carpal Tunnel Syndrome: A Prospective Randomized, Single-Blind Controlled Trial. Sci. Rep. 7 (1), 94. 10.1038/s41598-017-00224-6 28273894 PMC5427966

[B71] Wu Y. N. Liao C. H. Chen K. C. Chiang H. S. (2022). Dual Effect of Chitosan Activated Platelet Rich Plasma (Cprp) Improved Erectile Function after Cavernous Nerve Injury. J. Formos. Med. Assoc. 121 (1 Pt 1), 14–24. 10.1016/j.jfma.2021.01.019 33781654

[B72] Xing D. (2020). Study Characteristics and Tendency of Platelet-Rich Plasma Globally by Bibliometrics and Visualization Analysis [J]. Chin. J. Tissue Eng. Res. 24 (21), 3358–3362.

[B73] Xu Z. Yin W. Zhang Y. Qi X. Chen Y. Xie X. (2017). Comparative Evaluation of Leukocyte- and Platelet-Rich Plasma and Pure Platelet-Rich Plasma for Cartilage Regeneration. Sci. Rep. 7, 43301. 10.1038/srep43301 28265109 PMC5339695

[B74] Ye F. Li H. Qiao G. Chen F. Tao H. Ji A. (2012). Platelet-rich Plasma Gel in Combination with Schwann Cells for Repair of Sciatic Nerve Injury. Neural Regen. Res. 7 (29), 2286–2292. 10.3969/j.issn.1673-5374.2012.29.007 25538751 PMC4268730

[B75] Yin W. Xu H. Sheng J. Xu Z. Xie X. Zhang C. (2017). Comparative Evaluation of the Effects of Platelet-Rich Plasma Formulations on Extracellular Matrix Formation and the NF-Κb Signaling Pathway in Human Articular Chondrocytes. Mol. Med. Rep. 15 (5), 2940–2948. 10.3892/mmr.2017.6365 28339078 PMC5428536

[B76] Zhang S. Cao D. Xie J. Li H. Chen Z. Bao Q. (2019). Platelet-rich Fibrin as an Alternative Adjunct to Tendon-Exposed Wound Healing: A Randomized Controlled Clinical Trial. Burns. 45 (5), 1152–1157. 10.1016/j.burns.2019.01.007 30686693

[B77] Zhang Z. Yu B. Gu Y. Zhou S. Qian T. Wang Y. (2016). Fibroblast-derived Tenascin-C Promotes Schwann Cell Migration through β1-integrin Dependent Pathway during Peripheral Nerve Regeneration. Glia 64 (3), 374–385. 10.1002/glia.22934 26497118

[B78] Zhao T. Yan W. Xu K. Qi Y. Dai X. Shi Z. (2013). Combined Treatment with Platelet-Rich Plasma and Brain-Derived Neurotrophic Factor-Overexpressing Bone Marrow Stromal Cells Supports Axonal Remyelination in a Rat Spinal Cord Hemi-Section Model. Cytotherapy. 15 (7), 792–804. 10.1016/j.jcyt.2013.04.004 23731762

[B79] Zheng C. Zhu Q. Liu X. Huang X. He C. Jiang L. (2014). Improved Peripheral Nerve Regeneration Using Acellular Nerve Allografts Loaded with Platelet-Rich Plasma. Tissue Eng. Part. A. 20 (23-24), 3228–3240. 10.1089/ten.TEA.2013.0729 24901030 PMC4259182

[B80] Zheng C. Zhu Q. Liu X. Huang X. He C. Jiang L. (2016). Effect of Platelet-Rich Plasma (PRP) Concentration on Proliferation, Neurotrophic Function and Migration of Schwann Cellsin Vitro. J. Tissue Eng. Regen. Med. 10 (5), 428–436. 10.1002/term.1756 23723151

[B81] Zhu Y. Jin Z. Fang J. Wang J. Wang Y. Song Q. (2020). Platelet-Rich Plasma Combined with Low-Dose Ultrashort Wave Therapy Accelerates Peripheral Nerve Regeneration. Tissue Eng. Part. A. 26 (3-4), 178–192. 10.1089/ten.TEA.2019.0187 31516089

